# High-Density Lipoprotein in Metabolic Disorders and Beyond: An Exciting New World Full of Challenges and Opportunities

**DOI:** 10.3390/ph16060855

**Published:** 2023-06-08

**Authors:** Evangelia Zvintzou, Eva Xepapadaki, George Skroubis, Victoria Mparnia, Katerina Giannatou, Karim Benabdellah, Kyriakos E. Kypreos

**Affiliations:** 1 Department of Pharmacology, School of Medicine, University of Patras, Rio Achaias, 26500 Patras, Greece; 2 Morbid Obesity Unit, Department of Surgery, School of Medicine, University of Patras, Rio Achaias, 26500 Patras, Greece; 3 Department of Genomic Medicine, Pfizer-University of Granada-Andalusian Regional Government Centre for Genomics and Oncological Research (GENYO), PTS, Avda. de la Ilustración 114, 18016 Granada, Spain; karim.benabdel@genyo.es; 4 Department of Life Sciences, School of Sciences, European University Cyprus, 2404 Nicosia, Cyprus

**Keywords:** high-density lipoprotein, atherosclerosis, NAFLD, type 2 diabetes mellitus, morbid obesity, adipose tissue, multiple myeloma, gene-editing, pharmacology, pharmaceuticals

## Abstract

High-density lipoprotein (HDL) is an enigmatic member of the plasma lipid and lipoprotein transport system, best known for its ability to promote the reverse cholesterol efflux and the unloading of excess cholesterol from peripheral tissues. More recently, data in experimental mice and humans suggest that HDL may play important novel roles in other physiological processes associated with various metabolic disorders. Important parameters in the HDL functions are its apolipoprotein and lipid content, further reinforcing the principle that HDL structure defines its functionality. Thus, based on current evidence, low levels of HDL-cholesterol (HDL-C) or dysfunctional HDL particles contribute to the development of metabolic diseases such as morbid obesity, type 2 diabetes mellitus, and nonalcoholic fatty liver disease. Interestingly, low levels of HDL-C and dysfunctional HDL particles are observed in patients with multiple myeloma and other types of cancer. Therefore, adjusting HDL-C levels within the optimal range and improving HDL particle functionality is expected to benefit such pathological conditions. The failure of previous clinical trials testing various HDL-C-raising pharmaceuticals does not preclude a significant role for HDL in the treatment of atherosclerosis and related metabolic disorders. Those trials were designed on the principle of “the more the better”, ignoring the U-shape relationship between HDL-C levels and morbidity and mortality. Thus, many of these pharmaceuticals should be retested in appropriately designed clinical trials. Novel gene-editing-based pharmaceuticals aiming at altering the apolipoprotein composition of HDL are expected to revolutionize the treatment strategies, improving the functionality of dysfunctional HDL.

## 1. Introduction

Extensive clinical evidence indicates that low-plasma, high-density lipoprotein (HDL)-cholesterol (HDL-C) levels constitute an important risk factor in coronary heart disease (CHD) development and overall cardiovascular mortality [1]. Despite the original assumption that HDL-C levels are inversely correlated with cardiovascular (CVD) risk, recent epidemiological studies redefined this relationship, showing that it is “U-shaped” [2], with the optimal HDL-C levels in the range of 40–70 mg/dL for men and 50–70 mg/dL for women. As a result, although high HDL-C levels (>70 mg/dL) are associated with increased CVD risk, a substantial benefit is expected when HDL-C levels are raised from below 40 mg/dL for men or 50 mg/dL for women to levels up to 70 mg/dL.

As research in the field of lipoproteins progressed, it became apparent that in addition to HDL-C quantity, HDL particle functionality, which depends on its structural and functional characteristics, is also very important in cardioprotection. The functions of HDL may render it “antiatherogenic” or “proatherogenic” depending on the conditions [3,4]. More recent findings from mouse studies and clinical trials now indicate that HDL proteome affects its lipid cargo (lipidome) and both its functionalities [5,6]. It is quite possible that the qualitative characteristics of HDL are responsible for the U-shape correlation between HDL-C levels and atheroprotection, but this remains to be shown. The discovery of the U-shape relationship between HDL-C levels and coronary heart disease sparked recent intense interest in the field of atherosclerosis with many new clinical trials in progress, aiming at adjusting HDL-C levels within the proper therapeutic window.

At the molecular level, HDL particle metabolism starts with its biogenesis in the circulation where lipid-free apolipoprotein A1 (APOA1) interacts with lipid transporters ATP-binding cassette A1 (ABCA1) to form minimally lipidated discoidal HDL particles, which may further interact with ATP-binding cassette G1 (ABCG1) and the plasma enzymes lecithin:cholesterol acyl transferase (LCAT), cholesteryl-ester transfer protein (CETP), and phospholipid transfer protein (PLTP). Adding to the complexity of HDL metabolism, other apolipoproteins may also form HDL particles, independently of APOA1. Once formed in the periphery, HDL is taken up mainly by the liver via scavenger receptor class B member I (SRB1), completing the transfer of cholesterol from periphery to the liver, also known as “reverse cholesterol transport”.

## 2. HDL Proteome and Particle Functionality

Recent preclinical and clinical evidence established a relationship between HDL composition and function [3,5,6,7,8]. In addition to APOA1, many other apolipoproteins associated with HDL, with the two most abundant being APOA1 and apolipoprotein A2 (APOA2) [9,10,11,12]. Although APOA1 contributes to the formation of classical HDL particles and contributes to the protection from atherosclerosis [13,14], more recent data show that the anti-inflammatory and antioxidant properties of HDL are greatly affected by its overall apolipoprotein content [6,7,8]. Interestingly, different HDL particles carry different apolipoprotein cargo and perform different functions [3,5,6,7,8].

Indeed, experiments in mice that were deficient in both apolipoprotein E (APOE) and APOA1 (*Apoe*^−/−^ × *Apoa1*^−/−^ mice) and infected with a APOA1-expressing adenovirus indicate that APOA1-rich HDL (APOA1-HDL) is incapable of lowering the secretion of tumor necrosis factor (TNFa) by cultured RAW 264.7 macrophages, following stimulation with lipopolysaccharide (LPS) [6]. In contrast, APOE3-containing HDL (APOE3-HDL), isolated from the same mouse strain infected with an APOE3-expressing adenovirus, significantly reduced the secretion of TNFα by RAW 264.7 macrophages, showing that APOE-containing HDL (APOE-HDL), but not APOA1-HDL, is anti-inflammatory. Along the same line, APOA2-rich HDL (APOA2-HDL) isolated from wild-type mice infected with an APOA2-expressing adenovirus lowered TNFα production following LPS stimulation of macrophages, suggesting that APOA2-HDL is anti-inflammatory [7]. In contrast, when apolipoprotein C3 (APOC3)-containing HDL (APOC3-HDL) particles were tested, an excess release of TNFα was observed, suggesting a proinflammatory role of APOC3-HDL [8].

Similar to the anti-inflammatory property, the antioxidant property of HDL is also highly modulated by its apolipoprotein scaffold. The APOE3-HDL particles described above showed reduced antioxidant potential compared to APOA1-HDL particles [6]. However, APOC3-HDL and APOA2-HDL possessed significantly higher antioxidant capacity.

When cholesterol efflux was assessed, both APOC3- and APOA2-HDL did not enhance the ability of HDL to receive [^14^C]-cholesterol from cholesterol-laden RAW 264.7 cells, indicating reduced total cholesterol efflux capacity (CEC) [7,8].

In addition to apolipoproteins, HDL proteome includes many other protein components such as PLTP, LCAT, paraoxonase 1 (PON1), myeloperoxidase (MPO), serum amyloid A, (SAA), and platelet activating factor acetylhydrolase (PAF-AH), also known as lipoprotein-associated phospholipase A2 (Lp-PLA2). The presence of these proteins and their relative concentration on each HDL particle affect its functionality [1].

## 3. HDL Lipidome and Particle Functionality

A large volume of preclinical data indicate that HDL proteome defines to a great extent the amount and type of lipids associated with HDL particles [3]. Currently, ten different types of phospholipids and seven different types of sphingolipids are discovered in HDL lipidome [15]. Out of these lipids, phosphatidylcholine and sphingomyelin are the most abundant lipids present on the HDL particle surface [8,15]. Sphingosine 1-phosphate (S1P) is shown to mediated important antithrombotic and vasoprotective functions performed by HDL [16]. In addition, S1P promotes reverse cholesterol transport (RCT) and blocks low-density lipoprotein (LDL) oxidation and monocyte adhesion to the vascular wall [17]. The levels of S1P in HDL lipidome have been inversely correlated with CVD, acute myocardial infarction (MI), coronary artery disease (CAD), and ischemic heart disease [18,19].

HDL lipidome is also quite crucial for the antioxidant function of HDL particles. In particular, the amount and type of lipids present in HDL can impact the fluidity of its phospholipid layer and, thus, its capacity to receive and exchange lipids. Data suggest that the transfer of oxidized lipids from LDL to HDL requires a highly fluid phospholipid monolayer [20]. Lipids, which may turn this monolayer more rigid, limit the ability of HDL to receive oxidized lipids from LDL, thus reducing the LDL-oxidative status [21]. Such HDL particles have been reported in pathological conditions, where HDL is rich in triglycerides (TG) and has lower-than-expected levels of cholesteryl esters [22,23,24]. However, the optimal HDL lipid layer fluidity is not defined. An important parameter affecting the fluidity of the HDL phospholipid monolayer is the relative presence of mono- and poly-unsaturated fatty acids [25,26]. Increased levels of mono-unsaturated fatty acids are expected to reduce lipid packing and contribute to a higher membrane fluidity, while poly-unsaturated and saturated fatty acids may lead to a more rigid lipid layer. It should be noted, however, that the data from the eicosapentaenoic acid (EPA) vs. docosahexaenoic acid (DHA) comparison show that, in addition to the class of fatty acid, the number of carbon present is also an important determinant since longer or shorter carbon chains may influence the steric accommodation of monounsaturated lipids on the HDL phospholipid monolayer.

## 4. High-Density Lipoprotein and Plasma Triglyceride Levels

In general, plasma HDL-C levels show an inverse correlation with plasma triglyceride levels [27,28]. Previous data indicate that at the mechanistic level, the ability of exchangeable apolipoproteins to interact with ABCA1 and form HDL particles reduces the availability of these apolipoproteins for triglyceride-rich (TG-rich) lipoproteins such as very-low-density lipoprotein (VLDL) and chylomicrons [29]. These apolipoproteins include APOE, apolipoprotein C1 (APOC1), apolipoprotein C2 (APOC2), and APOC3, all of which are potent inhibitors of lipoprotein lipase when present in excess on TG-rich lipoproteins. Therefore, HDL may act as a buffer that prevents accumulation of excess plasma apolipoproteins on TG-rich particles. Under the condition when HDL formation is impaired, this HDL buffering capacity is reduced or even eliminated, resulting in abnormal apolipoprotein composition of VLDL, inhibition of triglyceride lipolysis, and hypertriglyceridemia [29].

The marked reduction in HDL formation found in patients with Tangier’s disease may also explain the mild to moderate hypertriglyceridemia of these patients [30,31,32]. In previous studies, structural analyses and post-heparin-lipolytic activities of VLDL isolated from Tangier’s disease patients showed that a lack of functional ABCA1 results in the abnormal apolipoprotein composition of VLDL, reduced reactivity of VLDL-triglycerides with plasma lipoprotein lipase (LPL), and hypertriglyceridemia [31,33].

## 5. High-Density Lipoprotein in Type 2 Diabetes Mellitus

In addition to atherosclerosis, in recent years, a bidirectional close correlation of type 2 diabetes mellitus (T2DM) with HDL-C levels and HDL particle functionality has been established [34]. T2DM, manifested by elevated blood glucose levels, is a major health problem worldwide, ranking among the top 10 causes of mortality in adults. Based on epidemiological evidence from the World Health Organization (WHO) in 2014, 422 million people with T2DM were reported, increasing the overall prevalence of this disorder from 4.7% in 1980 to 8.5% [35]. This prevalence is expected to rise further to 10.2% (578 million) globally by 2030 and 10.9% (700 million) by 2045 [36]. Insulin resistance occurs when pancreatic beta islets fail to produce and/or secrete sufficient insulin to fulfil metabolic needs, or when insulin-sensitive organs lose their capacity to respond to insulin stimulation [34]. Insulin resistance usually precedes the diagnosis of T2DM and is associated with a distinct type of dyslipidemia, characterized by elevated triacylglycerols (TAGs) and non-esterified fatty acids (NEFA), low HDL-C levels, and proatherogenic small dense LDL particles [34]. Insulin suppresses hepatic TG-rich lipoprotein production, and as a result, in insulin-resistant states, an increase in hepatic VLDL production and secretion is observed [34]. Plasma HDL-C levels and HDL particle functionality have been linked to glucose homeostasis by four main mechanisms, including peripheral insulin sensitivity, pancreatic beta-islet insulin secretion, non-insulin-dependent glucose uptake, and adipose tissue metabolic activation. Increasing the levels of plasma HDL-C and APOA1 improves glycaemic control by improving the antioxidant capacity and reverse cholesterol transport capacity of HDL [34]. Additionally, it was recently shown that the AMP-activated protein kinase (AMPK) pathway does not mediate APOA1-stimulated heart and skeletal muscle glucose uptake. The APOA1 gene mutation rs670 has been shown to increase HDL-C levels, insulin levels, and the homeostatic model assessment for insulin resistance (HOMA-IR) while simultaneously being associated with decreased insulin resistance [34].

**Figure 1:**
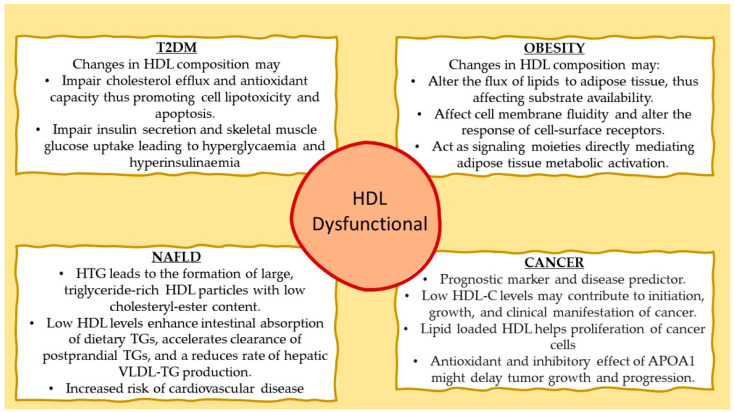
Proposed mechanisms involving dysfunctional HDL with type 2 diabetes mellitus (T2DM), obesity, nonalcoholic fatty liver disease (NAFLD), and cancer.

Recently, the causal relationship between T2DM and the structure of HDL was investigated in a preclinical study assessing the effects of APOA1 and LCAT deficiency in diet-induced obesity and glucose homeostasis in mice [25]. APOA1 deficiency, which is associated with the absence of classical APOA1-HDL particles, and LCAT deficiency, which is associated with the presence of mainly discoidal HDL particles, both lead to drastic alterations in the structure and composition of HDL. As a result, HDL from APOA1-null (*Apoa1*^−/−^) mice contained primarily APOE, and HDL from LCAT-null (*Lcat^−^*^/*−*^) mice contained mainly APOA2; both strains had significantly reduced HDL-C levels, although only *Apoa1*^−/−^ mice were presented with a T2DM phenotype because of the reduced ability of pancreatic β-islets to secrete insulin in response to glucose stimulation and the reduced skeletal muscle glucose uptake in response to insulin [25]. This finding was attributed to the beta cell membrane’s enhanced rigidity, a physicochemical characteristic linked to decreased activation of ion channels, a process crucial for insulin secretion. Similarly, skeletal muscles of only *Apoa1*^−/−^ mice showed reduced glucose uptake upon insulin stimulation [37]. These results strongly imply that the structure and function of HDL particles, rather than body fat accumulation, are the decisive parameters for glucose metabolic abnormalities, despite obesity being frequently referred to as the etiology of T2DM development in people with metabolic syndrome (Figure 1).

## 6. High-Density Lipoprotein and Adipose Tissue Metabolic Activity

The structural modifications of HDL particles in *Apoa1*^−/−^ and *Lcat^−/−^* mice described above correlated with characteristic metabolic aberrations. Both strains developed obesity following feeding with high-fat diet because of much-reduced non-shivering thermogenesis in their white adipose tissue (WAT), as documented by reduced mitochondrial uncoupling protein 1 (Ucp1) expression [25]. Similarly, *Apoa1*^−/−^ mice failed to induce WAT and brown adipose tissue (BAT) thermogenesis under cold exposure. Ectopic expression of APOA1 (i.e., formation of APOA1-HDL) in these mice using adenovirus-mediated gene transfer promoted metabolic activation of mitochondrial in BAT only when apolipoprotein E3 (APOE3-HDL) was virtually undetectable because APOE3 exerted an inhibitory effect on this process [25]. In contrast, stimulation of thermogenesis in WAT was subjected to a more complicated regulation that required the simultaneous presence of both APOA1-HDL and APOE3-HDL at a relative concentration ratio APOA1-HDL/APOE3-HDL of about 1.4, a typical ratio observed in wild-type C57BL/6 mice [25]. These data suggested a positive functional interaction between APOA1 and APOE3 in the activation of WAT.

Apolipoprotein E is another major apolipoprotein component of various lipoproteins, including HDL, which was recently shown to be crucial in WAT metabolic activation and maintenance of plasma glucose homeostasis [38]. Indeed, we found that APOE3 has an opposing dual effect on metabolic activation of visceral WAT mitochondria that is dependent on the tissue it is expressed in but independent of the ability of APOE3 to promote postprandial dietary lipid delivery to various tissues. Briefly, APOE3 expressed in the brain inhibited oxidative phosphorylation in visceral WAT mitochondria, leading to increased body weight and obesity in response to a Western-type diet. In contrast, hepatic APOE3 expression (i.e., APOE3 presence only in the periphery) triggered increased thermogenesis in visceral WAT mitochondria, leading to a lean phenotype [39]. Yet, it is still unclear exactly how hepatically or cerebrally produced APOE affects WAT metabolism. These findings constitute a major “paradigm shift” from the existing perception of *Apoe* when peripheral expression of APOE promotes obesity via receptor-mediated postprandial delivery of dietary lipids to WAT [39].

Despite a significant body of evidence, to this date, it is not clear how HDL may influence WAT and BAT mitochondrial metabolic activity. One possibility could be that APOA1-HDL and APOE-HDL modulate the delivery of lipids to these two subtypes of fat, thus affecting thermogenesis and oxidative phosphorylation by altering substrate availability. Another possibility could be that these lipoproteins have a direct effect on the biophysical characteristics of cellular membranes, thus effecting their fluidity, which in turn is important for the ability of cell-surface receptors to respond to external stimuli [25]. A third possibility is that APOA1-HDL and APOE-HDL could act as signaling moieties, through bioactive lipids present in their lipid cargo, that can directly impact the metabolic state of WAT and BAT (Figure 1).

## 7. High-Density Lipoprotein and Hepatic Triglyceride Deposition

In patients with hypertriglyceridemia, the transfer of triglycerides from triglyceride-rich lipoproteins to HDL particles, mediated by CETP, is enhanced [40], leading to the formation of large, triglyceride-rich HDL particles with low cholesteryl-ester content [41,42]. Hepatic lipase targets preferably these triglyceride-rich HDL particles and hydrolyzes their triglycerides, promoting HDL uptake by the liver. Although changes in the anti-atherogenic HDL subfractions have been linked to liver fat levels, this association does not necessarily imply a direct causal relationship [41]. There is proof that dysfunctional HDL particles present in the plasma of nonalcoholic fatty liver disease (NAFLD) patients increase their risk of cardiovascular disease [43,44]. In addition, the link between elevated post-prandial triglycerides and impaired vascular endothelial function is well-established [45]. Earlier research demonstrated that triglyceride enrichment of HDL may modify its anti-atherogenic properties, including anti-oxidative and anti-inflammatory functions, negatively affecting its capacity to protect the endothelium and regulate vascular reactivity [46,47]. However, little is known about the relationship between liver fat content and post-prandial HDL compositional and functional alterations (Figure 1).

Previous work showed that deficiency in APOA1 in mice impacted intestinal and hepatic TG metabolism. Specifically, deficiency in APOA1 resulted in enhanced intestinal absorption of dietary TGs, accelerated clearance of postprandial TGs and a reduced rate of hepatic very-low-density lipoprotein TG production [48]. These alterations in plasma TG metabolism resulted in pronounced hepatic TG deposition and disturbed hepatic histology in response to a high-fat diet [48]. Interestingly, administration of APOA1_Milano_, a gain-of-function mutant of APOA1, by adenovirus-mediated gene transfer in *Apoa1*^−/−^ mice reduced hepatic lipid deposition and improved hepatic architecture, reinforcing the notion that reduced plasma APOA1 levels are important regulators of NAFLD [48].

Similarly, deficiency in LCAT led to altered plasma TG metabolism [49]. *Lcat*^−/−^ mice were also found prone to hepatic lipid deposition. Adenovirus-mediated gene transfer of human *Lcat* in *Lcat*^−/−^ mice led to a significant improvement of hepatic lipid deposition [49], confirming that formation of mature spherical HDL particles is essential for physiological levels of hepatic triglycerides.

## 8. High-Density Lipoprotein and Cancer

The lipid and lipoprotein transport system plays a crucial role in the pathogenesis and progression of many types of cancer [50], such as colorectal cancer [51,52,53], lung cancer [54,55], breast cancer [56], ovarian cancer [57], thyroid cancer [58], gastric cancer [59], and multiple myeloma (MM) [60].

Recent data indicate that HDL-C levels are positively correlated with overall cancer survival [61]. Low HDL-C levels may contribute to the initiation, growth, and clinical manifestation of cancer. In 2010, Jafri et al. reported the existence of an inverse relationship between HDL-C and cancer incidence, independent of age, body mass index (BMI), LDL-cholesterol (LDL-C), gender, diabetes, and smoking [62].

In addition to plasma HDL-C levels, alterations in the structural and functional characteristics of HDL particles may also occur in patients with different cancer types. For example, small-diameter HDL particles were observed in colorectal cancer patients but not in healthy individuals, indicating that these particles may serve as an additional predictor of the disease [53].

To meet their needs for proliferation, cancer cells have a high demand for lipids and cholesterol. According to a recent publication, several proteins, including ABCA1, APOA1, APOE, apolipoprotein M (APOM), and SRB1, are involved in the influx and efflux of lipids in cancer cells [63]. Data from epidemiological and animal research point to a potential involvement of APOA1 in different cancer types [55,64]. Additionally, it was proposed that, in addition to its antioxidant activity in cancer [55], APOA1 might have an inhibitory effect on tumor growth and progression and could have therapeutic effects in the treatment of cancer (Figure 1) [65].

With respect to multiple myeloma, according to previously published data, alterations in lipoprotein metabolism may cause particular cellular and molecular processes that could affect the microenvironment of the bone marrow and the differentiation of adipocytes [66]. One such clinical investigation compared plasma HDL-C, LDL-C, TGs, and total cholesterol levels of patients with MM to those of healthy subjects at various periods of the disease, including the time of diagnosis, throughout the active phase, and during disease remission. The data revealed that there were no stage-dependent differences in the levels of LDL-C and total cholesterol between the groups; however, HDL-C levels were lower in MM patients than in healthy participants, but only when the disease was active. Moreover, patients with MM had greater triglyceride levels than healthy controls both at the time of diagnosis and during the active period of the disease. However, during disease remission, the blood lipid levels were comparable between MM patients and controls, demonstrating a link between periods of active disease and changes in the levels of triglycerides and plasma lipoprotein cholesterol [67].

In another study, Yavasoglu et al. measured the serum lipid levels of 102 MM patients at various stages and types of the disease and found differences across the three MM stages according to the International Staging System (ISS) [68]. Total cholesterol levels were significantly lower in Stage II and III patients compared to healthy participants. Interestingly, however, HDL-C and LDL-C levels were much reduced in Stage III and II subjects, respectively, compared to healthy controls. However, plasma triglyceride and VLDL-cholesterol (VLDL-C) levels were similar between the two stages [68].

The prognostic significance of plasma components including APOA1, apolipoprotein B (APOB), total cholesterol, TG, HDL, and LDL for the various stages of the disease was assessed in 2019, according to the ISS, in a study involving 307 patients with MM [69]. Only those with MM who expressed higher levels of APOA1 had longer overall survival, progression-free survival, and cause-specific survival, according to the results. Despite their lower levels in the late ISS stage, other lipid metabolism indicators such as APOB, total cholesterol, and LDL-C did not correlate statistically significantly with either progression-free survival or overall survival. Last but not least, there were no variations in serum TG levels between ISS stages [69].

Recent studies have shown that morbid obesity is a significant risk factor for cancer [70], with 13 different cancer forms, including MM, being etiologically linked to the obese phenotype [71]. Other factors that contribute to diet-induced obesity include APOA1, the most abundant protein of HDL, APOE, an important protein of HDL/LDL/VLDL, and the functional ligand of an LDL receptor [39,72]. Hence, it is not irrational to hypothesize that the HDL particle composition may have an impact on the events leading to the onset and progression of MM.

In addition, it is worth mentioning that the composition and function of the cellular membrane could be, at least in part, involved in the development of MM. By delivering lipids to peripheral tissues, as in the case of chylomicron remnants, VLDL, and LDL, or by removing cholesterol and phospholipids from peripheral tissues, as in the case of HDL, serum lipoproteins could alter biophysical characteristics of cell membrane that could influence the cellular response to cancerous stimuli [60].

## 9. Current State of the Art in HDL Pharmaceuticals

In clinical trials, a wide range of pharmacological agents for increasing plasma HDL-C levels have been investigated (Table 1). The ineffectiveness of many CETP inhibitors such as torcetrapib (ClinicalTrials.gov: NCT00134264 (accessed on 28 May 2023)), dalcetrapib (ClinicalTrials.gov: NCT00658515 (accessed on 28 May 2023)), and evacetrapid (ClinicalTrials.gov: NCT01687998 (accessed on 28 May 2023)) [73] led to early serious concerns about their therapeutic significance [74]. Unfortunately, all those older trials were designed on the now outdated perception that the higher the HDL-C levels the better the protection from coronary heart disease and mortality. We now know that HDL-C levels and cardiovascular morbidity and mortality show a U-shape relationship with optimal HDL-C levels from 40–70 mg/dL for men and 50–70 mg/dL for women. Thus, the dose of CETP inhibitor used in those trials had, as a result, the massive elevation of HDL-C levels from around 20 mg/dL to over 80 mg/dL, a concentration range that is also associated with a very significant and even higher risk for morbidity and mortality. In other words, these poorly designed trials skipped the optimal HDL-C concentration range linked with the lowest risk of CVD and all-cause mortality. Therefore, the concept of CETP inhibition should be tested again but this time with a goal to raise low or very low HDL-C levels to the recommended range of 50–70 mg/dL for women and 40–70 mg/dL for men. This may be the case with obicetrapib, where low doses of up to 10 mg, much lower compared to other CETP inhibitors, were put to test (ClinicalTrials.gov: NCT01970215 (accessed on 28 May 2023)). These low doses of obicetrapib raised HDL-C levels from 24 mg/dL to a maximum of 65 mg/dL either as monotherapy or in combination with statins. Additionally, obicetrapib raised APOA1 by 50–60% and decreased APOB and lipoprotein (a) (Lp(a)) levels by 30–50% and 30%, respectively, either alone or on top of statin therapy [75]. It should be noted that statins increase Lp(a) levels [76]. Currently, obicetrapib is in a phase III clinical trial (ClinicalTrials.gov: NCT05202509 (accessed on 28 May 2023)) for the evaluation of its effect in patients with cardiovascular disease, and the results are expected by the end of 2026.

In addition to raising HDL-C levels, most recent studies have focused on altering the HDL proteome and lipidome in an effort to improve particle functionality. The design and development of reconstituted HDL (rHDL) particles, which resemble pre-β HDL, is one such strategy. These particles may play a significant role in the prevention of atherosclerosis by promoting ABCA1-mediated cholesterol efflux from lipid-rich macrophages. Three different types of APOA1 have been used in the rHDL manufacturing process: recombined human APOA1 purified from bacterial cultures, synthetic APOA1 peptides, and pure non-lipidated APOA1 isolated from human HDL particles [77]. The first human recombinant rHDL, apoA-1M ETC-216, apoA-1M, and MDCO-216 trials were discontinued because of adverse events and lack of efficacy in inducing regression of atherosclerosis when administered on top of statin therapy, respectively [78,79]. Additional research revealed that APOA1 mimetic peptides might enhance cholesterol efflux, reduce lipoprotein oxidation, and promote the formation of pre-β HDL particles, making them a promising novel therapeutic strategy for atherosclerosis [80]. The most extensively studied APOA1 mimetic peptide, D-4F, was shown to bind to oxidized lipids more effectively than endogenous APOA1 [81]. D-4F was shown to lower the HDL inflammatory index in the first-ever multiple-dose, randomized controlled trial in high-risk patients [82]. However, more recent genome-wide association studies do not support the idea that raising APOA1 levels in circulation may be the most effective method for preventing CVD [83].

The effectiveness and safety of several rHDL particles, including CER-001 (produced recombinant human APOAI and sphingomyelin), CSL-111 (produced by native APOAI and phospholipids), and CSL-112 (produced by reconstituted human-plasma-derived APOAI and phosphatidylcholine) have been examined in clinical trials. Results from the CARAT clinical trial (ClinicalTrials.gov: NCT02484378 (accessed on 28 May 2023)) failed to demonstrate regression of atherosclerosis in patients with ACS already receiving statin therapy after 10 weekly infusions of CER-001 [84]. On the other hand, a single infusion of CSL-111 decreased the lipid content in the atherosclerotic plaque compared to placebo in a randomized controlled trial involving 20 patients (11 with a history of confirmed CAD on aspirin and 18 on statins) [85]. However, results from the ERASE trial (ClinicalTrials.gov: NCT00225719 (accessed on 28 May 2023)) showed that CSL-111 was not able to reduce atherosclerotic plaque volume and led to elevated levels of liver enzymes leading to discontinuance of CSL-111 development [86]. The most recent formulation of human APOAI, CSL112, was shown to be well-tolerated in human trials (ClinicalTrials.gov: NCT02108262 (accessed on 28 May 2023)) [87], opening the road to a big phase III clinical trial, AEGIS-II (ClinicalTrials.gov: NCT03473223 (accessed on 28 May 2023)). AEGIS-II is designed to assess the efficacy and safety of CSL112 and is expected to be completed by the end of 2023.

**Table 1:** Summary of the main HDL-C pharmacological agents currently available or under development.

Agent	Trial Status	Clinical Outcomes	Reference and NCT
Obicetrapib	Active, recruiting	Significant increase in HDL-C levels from 24 mg/dL to a maximum of 65 mg/dL. Significant increase in APOA1 by 50–60%. Significant decrease in APOB levels by 30–50% and Lp(a) levels by 30%.	[75] NCT05202509
Niaspan	Terminated (2012)	Significant increase in HDL-C levels by approximately 30%. Significant decrease in TG levels by 30%, LDL-C levels by 14%, (Lp(a)) levels by 32% and APOB levels by 9–39%.	[88] NCT00120289
D-4F	Completed (2017)	Binds to oxidized lipids more effectively than endogenous APOA1 and lowers the HDL inflammatory index.	[81,82].
CER-001	Completed (2016)	Failed to demonstrate regression of atherosclerosis in patients with ACS already receiving statin therapy after 10 weekly infusions of CER-001.	[84] NCT02484378
CSL-111	Completed (2008)	Did not reduce atherosclerotic plaque volume and led to elevated levels of liver enzymes. Its development was discontinued.	[86] NCT00225719
CSL-112	Active, not recruiting	Well-tolerated in human trials. Increased HDL cholesterol efflux capacity. Currently assessing efficacy and safety. Results are expected by the end of 2023.	[87] NCT03473223

Our recent observation that different apolipoproteins recruit different types of lipids on HDL and both result in distinct particle properties [6,7,8] may explain why recombinant APOA1-containing HDL particles were not found effective in treating atherosclerosis. Indeed, in our studies, APOA1-HDL could effectively promote cholesterol efflux but was proinflammatory [6].

Currently, the only approved medicine capable of increasing HDL-C levels by approximately 30% is niacin. In addition, it decreases TG by 30%, LDL-C by 14%, lipoprotein (a) (Lp(a)) by 32%, and APOB by 9–39% [88]. Unfortunately, however, the AIM-HIGH (ClinicalTrials.gov: NCT00120289 (accessed on 28 May 2023)) and the HPS2-THRIVE (ClinicalTrials.gov: NCT00461630 (accessed on 28 May 2023)) trials failed to demonstrate the benefit of niacin on cardiovascular morbidity and mortality [89]. This is a highly controversial conclusion given that the level of the quality of clinical evidence from these trials is very low, when evaluated based on the principles of GRADE (grading of recommendations, assessment, development, and evaluations) [1]. The favorable effect of niacin on the lipoprotein profile could be proven beneficial in properly designed clinical trials addressing the weaknesses of AIM-HIGH and HPS2-THRIVE. For example, niacin could reduce residual cardiovascular risk in patients with hypertriglyceridemia (a population similar to those tested in REDUCE-IT trial, Clinicatrials.gov: NCT01492361 (accessed on 28 May 2023)) [1].

## 10. Opportunities for Novel Pharmaceuticals in the Gene-Editing Era

**Figure 2:**
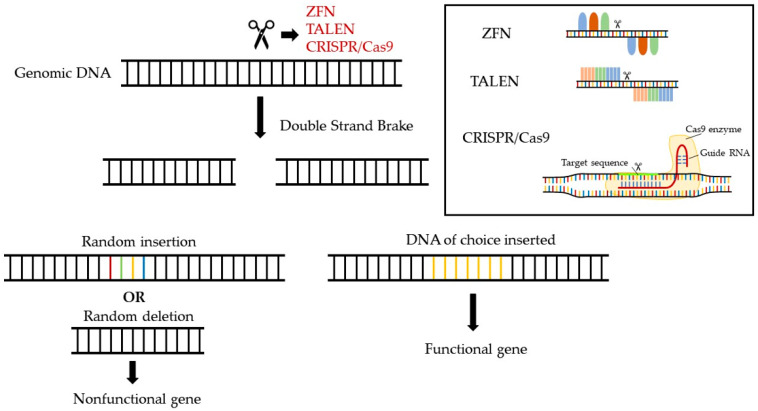
Gene-editing strategies currently available for the cure of genetic and hereditary disorders.

Even though today there are limited strategies using classical medicinal products to increase low HDL-C levels (hypoalphalipoproteinemia) and improve the functionality of HDL particles, the development of advanced-treatment medicinal products (ATMP) holds a great future promise for the pharmacology of HDL. ATMPs are medicines for human use that are based on genes, tissues, or cells and may be classified in four distinct subcategories: (a) gene therapy medicines that deliver genes as a means of treating a genetic disorder, (b) somatic-cell therapy medicines that deliver cells or tissues that have been manipulated ex vivo to perform the anticipated therapeutic actions, (c) tissue-engineered medicines that deliver cells or tissues that have been modified so they can be used to repair, regenerate, or replace human tissue, and (d) gene-editing medicines that perform highly specific alternations in the human genome, aiming at silencing disease-triggering genes, correcting disease-causing mutations, replacing defective genes, providing epigenetic modifications, or modifying the levels of gene expression [90]. Such novel pharmaceuticals offer groundbreaking new opportunities for the treatment of disease and injury. Gene editing is a highly promising strategy with a great potential to transform medicine by providing precise and personalized therapies for a wide range of genetic disorders [91,92]. Five major gene-editing approaches have been developed over the past decades and are currently explored for potential therapeutic applications: Clustered Regularly Interspaced Short Palindromic Repeats (CRISPR), Transcription Activator-Like Effector Nucleases (TALENs), Zinc finger nucleases (ZFNs), base editing, and RNA editing (Figure 2) [93,94]. The CRISPR system is a broadly employed gene-editing method that uses a guide RNA (gRNA) to direct a Cas nuclease to any specific DNA sequence, enabling the insertion, deletion, or replacement of specific genetic material into the host genome. Similarly, TALENs and ZFNs bind to specific DNA sequences and enable the cutting of the DNA by a specific nuclease. Each zinc finger recognizes a 3-base-pair DNA. Several zinc fingers can be combined in a modular way to recognize longer DNA sequences. TALENs, on the other hand, are composed of a succession of identical repeats, each of which contains a highly conserved 33- to 35-amino-acid sequence that allows DNA binding. The amino acid sequence within each repeat is known as the “repeat-variable diresidues” (RVDs) and is capable of precisely recognizing one single base pair. During the last years, researchers have developed base editors, adapted from the CRISPR/Cas9 system, to change individual nucleotides in a genome without cutting the DNA, thus increasing the accuracy and reducing the number of non-specific DNA modifications. Several types of base editors that have been developed include (a) cytidine deaminase base editors (CBEs), which shift a cytosine base (C) to a uracil base (U), which is then modified by the cell’s DNA repair machinery to a thymine base (T), and (b) adenine base editors (ABEs), which convert an adenine base (A) into a guanine base (G) in DNA [93,95]. Finally, RNA editing is one of the most recent updates of genome editing [96]. In particular, the CRISPR/Cas13 system allows for precise modifications to RNA sequences, further increasing the spectrum of potential applications [97,98,99].

### 10.1. Examples of Gene-Editing-Based Pharmaceuticals in Cardiovascular Diseases

Gene editing has emerged as a promising strategy for modifying the components of the plasma lipoprotein metabolic system, including HDL disorders, aiming at treating atherosclerotic cardiovascular disease. One such paradigm is the silencing of proprotein convertase subtilisin/kexin type 9 (PCSK9) gene encoding for proprotein convertase subtilisin/kexin type 9 protein, which reduces the levels of LDL receptors (LDLR) on the surface of a variety of cells, such as mice hepatic cells in vivo and human hepatoma cells (HepG2 and HuH7) or human embryonic kidney cells (HEK-293 cells) in vitro, leading to increased LDL-C in circulation [100]. Mutations in this gene can lead to high LDL-C levels and increased risk of heart disease. Interestingly, PCSK9 silencing significantly increases the cholesterol efflux capacity of HDL particles through the action of lipid transporter ABCG1 and aqueous diffusion pathways and reduces cellular cholesterol content in patients with familial hypercholesterolemia [101]. Several approaches based on the CRISPR-Cas9 system have been used to edit the PCSK9 gene in animal models, resulting in lower LDL cholesterol levels and reduced atherosclerosis. Li et al. used an adeno-associated virus (AAV) vector to deliver a Cas9-gRNA complex targeting the PCSK9 gene to the liver of mice. They observed a significant reduction in PCSK9 expression and circulating PCSK9 levels, as well as a reduction in LDL cholesterol levels in blood [102]. More recently, Lee et al. utilized liposomal nanoparticle (LNP)-mediated delivery to target the liver of cynomolgus monkeys and base-editing technology to make a single A-to-G base change at a specific site in the PCSK9 gene. This led to a successful disruption of PCSK9 protein expression, leading to an effective reduction in the LDL-C levels in the blood of the nonhuman primates [103].

Another paradigm of a candidate gene is APOC3. This apolipoprotein is a protein component of HDL that affects particle functionality, as described above. In addition, APOC3 plays a role in the metabolism of lipids, including triglycerides. Dysfunctional HDL and elevated levels of APOC3 have been associated with an increased risk of cardiovascular diseases such as atherosclerosis, coronary artery disease, and stroke [104,105,106]. Several studies have suggested that targeting APOC3 could be a promising strategy for reducing the risk of cardiovascular disease. For example, in a recent clinical trial, an antisense oligonucleotide drug targeting APOC3 significantly reduced triglyceride levels and the risk of cardiovascular events in patients with hypertriglyceridemia [107]. Similarly, preclinical studies have shown that gene-editing approaches targeting APOC3 can reduce the expression of APOC3 and lower triglyceride levels in hamsters and rabbits [108,109].

Similarly, ANGPTL3 (angiopoietin-like 3) is a gene that encodes a protein that regulates lipid metabolism in the body. ANGPTL3 has been shown to play a key role in the lipoprotein system by increasing circulating levels of triglycerides and LDL-C, which are two major risk factors for cardiovascular disease [110]. Contrasting the prevailing view that triglyceride levels correlate inversely with HDL-C level, ANGPTL3 also increases HDL-C levels because of reduced activity of LPL and endothelial lipase (EL) [111]. Recent studies have shown that inhibiting ANGPTL3 activity can reduce the risk of cardiovascular disease; an optimized LNP (lipid nanoparticle) system has been developed for delivering CRISPR/SpCas9 mRNA and ANGPTL3-targeting guide RNA to liver cells of wild-type C57BL76 to inhibit ANGPTL3 activity and reduce the risk of cardiovascular disease. The study showed that the LNP system was able to efficiently deliver the CRISPR components to liver cells, resulting in targeted cleavage of the Angptl3 gene and inhibition of ANGPTL3 protein production. The study also showed that treatment with the CRISPR/SpCas9 mRNA and ANGPTL3-targeting guide RNA LNP system reduced lipoprotein cholesterol and triglyceride levels with no evidence of off-target mutagenesis [112].

### 10.2. Challenges in the Development of Gene-Editing Pharmaceuticals

CRISPR/Cas9 and other gene-editing tools hold great promise for the treatment of various types of genetic dyslipidemias and associated cardiovascular disorders. However, several major challenges are still to be addressed. The first is the safety of these strategies, which refers to the specificity of the editing process. To this date, off-target effects, unintended mutations, and chromosomal aberrations may result from gene-editing strategies, posing significant obstacles in the translation of preclinical strategies into clinical applications. The second major challenge is the identification of the proper delivery system for the targeted delivery of the gene-editing machinery. This is of paramount importance since selecting the proper delivery vehicle will limit gene editing only to appropriate cells and tissues in a safe and efficient manner and without causing unwanted immune responses or other adverse effects [113]. Luckily, for disorders of the lipoprotein transport system, hepatocytes are the prime target, which is easily attainable with current delivery methods (i.e., viral systems and lipid nanoparticles).

### 10.3. Opportunities for Gene-Editing Therapies for Treating Low and Dysfunctional HDL

Previous studies by us and others showed that the apolipoprotein cargo of HDL dictates its lipid cargo and both the functionalities of the particle [86,87,88]. Therefore, novel pharmaceuticals based on gene editing could alter apolipoprotein composition of dysfunctional HDL particles and may be the most suitable treatment for the restoration of physiological HDL function. One such gene target could be the gene encoding for ABCA1. Loss-of-function mutation in the gene encoding ABCA1 causes the autosomal recessive genetic disorder Tangier disease associated with very low HDL-C levels and severe hypertriglyceridemia. There is currently no cure for Tangier disease, but treatment focuses on managing symptoms and reducing the risk of complications. Therefore, CRISPR/Cas9 gene editing may have the potential to correct the ABCA1 gene mutations in vivo, restoring the function of the lipid transporter. Similarly, it has been shown that the A164S mutation in human APOA1, present at a frequency of 1:500, is associated with increased hazard ratios for ischemic heart disease myocardial infarction and total mortality of 3.2 [95%CI:1.6–6.5], 5.5 [95% CI: 2.6–11.7], and 2.5 [95% CI: 1.3–4.8], respectively, in heterozygotes compared with noncarriers. Conversion of 164S to the wild-type 164A may be another suitable target. Along the same line, other loss-of-function mutations in APOA1, such as c.409G > T, which are associated with familial HDL deficiency, may be suitable candidates for this type of pharmacological intervention [114]. Thus, the CRISPR/Cas-based pharmaceuticals could be used to alter the functionality of APOA1 and potentially other apolipoproteins (APOA2, APOC3) and improve the antiatherogenic functions of HDL.

## 11. Conclusions

To this date, the role of HDL in human health remains enigmatic. Preclinical data, as well as clinical evidence, support that low HDL-C levels remain a significant risk factor for atherosclerosis, as well as for T2DM, morbid obesity, and NAFLD. Surprisingly, other conditions that appear unrelated may also be impacted by HDL-C levels and particle functionality. Indeed, all data suggest that in addition to the quantity of HDL-C, the quality of HDL particles appears to be crucial and should be considered.

**Figure 3:**
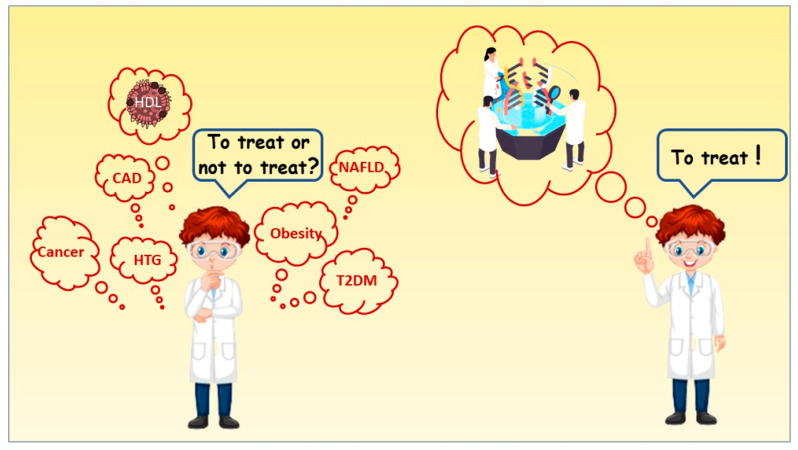
Despite previous speculations that HDL-C levels are irrelevant in clinical practice, current evidence supports that raising low HDL-C levels within the optimal range and improving the functionality of dysfunctional HDL are expected to have significant benefit to the prevention and treatment of multiple metabolic disorders.

Based on the existing body of evidence, raising HDL-C levels either with classical medicines or ATMPs is expected to have a beneficial effect on plasma triglyceride levels, reducing the residual risk associated with the use of LDL-lowering therapies [115]. Moreover, increasing HDL-C levels within the physiological may benefit patients with T2DM and morbid obesity (Figure 3).

Although in atherosclerotic cardiovascular disease, HDL-mediated reverse cholesterol transport along with its antioxidant and anti-inflammatory properties, for example, may explain its beneficial role, it remains unclear how this intriguing lipoprotein impacts conditions such as pancreatic β-cell secretory capacity, skeletal muscle insulin sensitivity, and adipose tissue mitochondrial metabolic activity. Initial evidence suggests that changes in cell membrane fluidity brought about by HDL may play a role in the later conditions [26] in a fashion similar to icosapent ethyl (IPE). However, other possibilities exist, and the precise mechanistic details remains to be elucidated.

## Data Availability

Data sharing not applicable.
